# The Role of Phosphatidylinositol Mannosides in the Serological Diagnosis of Mycobacterial Infections

**DOI:** 10.3390/vetsci6040091

**Published:** 2019-11-13

**Authors:** Ad P. Koets, Marielle H. van den Esker, Karel Riepema, Douwe Bakker

**Affiliations:** 1 Department of Bacteriology and Epidemiology, Wageningen Bioveterinary Research, Houtribweg 398221 RA Lelystad, The Netherlands; marielle.vandenesker@wur.nl (M.H.v.d.E.); karel.riepema@wur.nl (K.R.); 2 Department of Farm Animal Health, Faculty of Veterinary Medicine, Utrecht University, Yalelaan 73584 CL Utrecht, The Netherlands; 3 Independent Researcher, 8212 AM Lelystad, The Netherlands; douwe.bakker@kpnmail.nl

**Keywords:** *Mycobacterium avium* subsp. *paratuberculosis*, *Mycobacterium bovis*, glycolipid, phosphatidylinositol mannosides, cattle, tuberculosis, paratuberculosis, diagnosis

## Abstract

Accurate diagnosis of mycobacterial infections, such as bovine tuberculosis and paratuberculosis, remains challenging. Available direct diagnostic tests aimed at detecting the pathogen are highly specific but lack sensitivity, depending on the stage of infection and the prevalence of infection in a population. The sensitivity of indirect diagnostic assays that measure the host immune response to infection is similarly affected by disease characteristics. The choice of antigen used to detect a host response to infection has a critical impact on test sensitivity and specificity. Many indirect tests rely on crude antigen preparations and cell-free extracts, of which the production is poorly standardized. Moreover, these preparations contain ample uncharacterized cross-reactive compounds. To enhance serological test specificity, existing assays depend on the pre-treatment of samples and a relatively high cut-off value, that in turn influences test sensitivity. Research therefore focuses on the identification of more specific, defined antigens to improve diagnostics. In the current study, we extracted phosphatidylinositol mannosides (PIMs) and investigated their potential use in antibody-based tests. Our results demonstrate that specific IgG class antibodies are generated against PIMs in cows, but this is unrelated to tuberculosis or paratuberculosis infection status, making these antigens unsuitable for diagnostic applications. In addition, we demonstrate that PIMs are widely present in crude antigen preparations and in serum pre-absorption buffer. Our results indicate that PIMs are cross-reactive compounds with immunodominant B cell epitopes that could impair serological test specificity.

## 1. Introduction

Tuberculosis caused by *Mycobacterium bovis* (MB) infection remains a major problem in cattle and other ruminants in various countries around the world, such as the United Kingdom, Ireland, New Zealand, India, and Ethiopia [1]. In North America and parts of Europe, e.g. The Netherlands, countries introduced successful MB eradication campaigns and have been declared MB free, based on a very low prevalence. However, these countries still have to maintain active surveillance programs to safeguard this official tuberculosis free status. In both scenarios, adequate diagnostic tools are essential in the control of MB and need to take into account the (endemic) presence of non-tuberculous mycobacteria (NTM). While most of these NTM are non-pathogenic in healthy individuals, they can immunologically sensitize hosts [2]. In addition, some NTM species, and in particular *Mycobacterium avium* subsp. *paratuberculosis* (MAP), are ruminant pathogens causing severe disease [3]. MAP is the etiological agent of paratuberculosis, and is endemic worldwide. Control and eradication of paratuberculosis is difficult due to the long, subclinical lag-phase in which infection is not yet recognized but bacterial shedding already intermittently occurs [4]. This can lead to a rapid but unnoticed spreading within herds, affecting many animals and causing substantial economic impact.

The accurate diagnosis of mycobacterial infections like tuberculosis and paratuberculosis in ruminant and non-ruminant species remains challenging. For both diseases, the currently available direct diagnostic assays aimed at the detection of the respective pathogens are highly specific but lack sensitivity, depending on stage of infection and prevalence of infection in a population [5]. The sensitivity of the available indirect diagnostic tests that measure the host immune response to infection, such as antibody detecting enzyme-linked immunosorbent assays (ELISAs) and T cell assays (e.g., the intradermal tuberculin assay and the interferon gamma release assay), are similarly affected by disease characteristics. Additionally, the choice of antigen used to detect a host response to infection has a critical impact on test specificity, especially since sensitization to cross-reactive antigens occurs commonly due to the ubiquitous nature of environmental mycobacterial species [6].

Many of the indirect tests currently available rely on crude, partially, or ill-defined antigen preparations from mycobacterial cultures, like tuberculins or cell-free extracts, of which the production is poorly standardized [5]. Besides this, these preparations contain ample cross-reactive compounds that affect test specificity [6,7]. Prevailing indirect diagnostic assays therefore depend on comparative (skin) tests, relatively high cut-off values, and serum pre-absorption to enhance specificity. With respect to ruminant paratuberculosis, important improvements have been made by pre-absorbing serum with a specialized absorption buffer containing, for instance, a *Mycobacterium phlei* extract [8,9,10]. The number of available serological tests for bovine tuberculosis is much more limited, and serum pre-absorption has not been extensively studied [11]. However, from the studies regarding serodiagnosis of paratuberculosis it has become clear that increasing specificity through serum pre-absorption has a negative effect on test sensitivity, indicating the presence of immunodominant cross-reactive antigens [12]. The nature of these cross-reactive antigens has not been studied in detail, but the need for more specific and well standardized antigens is evident.

Potential antigenic candidates include glycolipids (GL), which comprise a substantial part of the mycobacterial cell wall. Several types of GL have been characterized, including the major lipids phosphatidylinositol mannosides (PIM), lipomannan (LM), and lipoarabinomannan (LAM) [13]. These GL are non-covalently attached to the inner or outer cell membrane by a phosphatidylinositol (PI) anchor. Biosynthesis of GL occurs in an ordered way: first, PI, consisting of a diacylglycerol backbone attached to an inositol ring, inserts into the membrane. Subsequently, PIM is synthesized by the addition of one to six mannose residues to carbon positions 2 and 6 of the inositol moiety by two mannosyltransferases, PimA and PimB [14]. PIM can be acylated by AcylT at the C6 position of the mannose ring, or at the C3 of the inositol ring. The degrees of mannosylation and acylation vary, but Ac_1_PIM_2_, Ac_2_PIM_2_, and AcPIM_6_ are the most prevalent PIM species [15]. PIMs form the basis of other GL, and LM and LAM are heavily glycosylated forms of PIM [16].

GLs, and especially PI and PIM, constitute a major fraction of the cell wall: in MB, around 56% of all phospholipids are made up by PI and PIM [17]. PIMs are important virulence factors that are required for host cell internalization, as well as intracellular trafficking and the biogenesis of phagosomes and their fusion with endosomes [18,19,20]. Moreover, it has been shown that PIMs are dominant non-peptidic mycobacterial antigens that are recognized by, e.g., TLR2, CD1, and the C-type lectins mannose receptors DC-SIGN and DCAR [21,22,23,24]. Thereby, these GL exert immunomodulatory effects on both the innate and adaptive immune system, which enhances long-term intracellular survival by inhibiting phagosome maturation, apoptosis, and cytokine production [25].

The antigenic surface-exposed properties of GL suggest their potential diagnostic value. Humans exposed to or infected with mycobacteria show high antibody responses to PIM and LAM in serodiagnostic tests [26,27], while ruminants infected with mycobacteria react to several GL, including LAM [28,29]. However, research focusing on the serodiagnostic potential of PIMs in ruminants is so far lacking. In the current study, we therefore evaluated immunological responses to PIM antigens with the aim of improving mycobacterial serodiagnostics. Our results indicate that PIM specific IgG class antibodies are generated in ruminant hosts. However, this is irrespective of the infection status, which makes these antigens unsuitable as diagnostic targets. In addition, we detected PIMs to be present in a number of frequently used antigenic preparations, including the ones used in pre-absorption buffer, indicating that these GL could contribute to non-specific serological cross-reactions.

## 2. Materials and Methods

### 2.1. Serum Samples

Archived serum samples from cattle present in the Wageningen Bioveterinary Research and Utrecht University biobanks were analyzed. The serum samples were obtained and archived between 1999 and 2006 from cattle enrolled in three separate experimental MAP or MB infection trials, as well as from diagnostic samples of naturally MAP and/or MB infected cattle. A summarizing overview of the samples used in the current study is presented in Table 1. The numbers 1, 2, 3, 4, and 5 represent samples from experimental infections. Details of two of the three experimental trials were reported previously in detail, as indicated (items 1, 2, and 3). The cattle from group 4 were experimentally infected with MB strain AN5, the animals in group 5 were uninfected control animals.

### 2.2. Statement of Ethical Approval

The animal experiments described in this study were performed in strict accordance with the provisions of the European Convention for the protection of vertebrate animals used for experimental and other scientific purposes (86/609 EG). The animal experiments were approved by the Animal Welfare Body of WBVR (numbers 1 (permit number 299-47053-07/99-01), 4, and 5 (permit number 821-47302-00/01-01)) and the Animal Welfare Body of Utrecht University (numbers 2 and 3, permit number 0202.0806) in accordance with the Dutch regulations on animal experimentation. Samples belonging to items 6, 7, 8, and 9 originated from field samples taken by veterinarians for diagnostic purposes and submitted to WBVR between 1999 and 2006 for (statutory) diagnostic testing.

### 2.3. Mycobacterial Antigen Preparations

Commercially available avian and bovine purified protein derivative (PPD) tuberculins, with a respective potency of 2500 IU and 3000 IU per 0.1 mL, were purchased from Thermo-Fisher Scientific (Lelystad, The Netherlands). Bovine PPD tuberculin (PPD-B) was produced from a culture of MB strain AN5 and avian PPD tuberculin (PPD-A) was produced from *Mycobacterium avium* subsp. *avium* (MAA) D4ER, both according to the procedures described in the European Pharmacopeia.

A large volume of the pre-absorption buffer routinely supplied with a commercially available paratuberculosis ELISA kit (ID Screen Paratuberculosis Indirect, ID Vet, Montpellier, France) containing *M. phlei* antigens to enhance assay specificity by limiting cross-reactions with mycobacteria other than MAP was a generous gift from the supplier.

### 2.4. Mycobacterial Cultures used for Antigen Preparations

Mycobacterial strains MB strain AN5, MAA D4, MAP 316F, and four MAP isolates (2148, 2869, 8479, 441,449 from cows with clinical signs of paratuberculosis) were cultured as pellicles in a stationary culture or in shaking cultures using Watson-Reid liquid medium (WR-medium) [32]. The pH was set at 5.7 using liquid ammonia 25% *w/v* (Merck, Darmstadt, Germany). For culture of MAP strains, 1 mg of mycobactin (Allied Monitor, Fayette, USA) was added per liter. Media were sterilized by autoclave for 30 min at 110 °C. Cultures were harvested after 8 to 16 weeks of incubation.

### 2.5. Isolation of Polar (glyco)lipids

**Figure 1:**
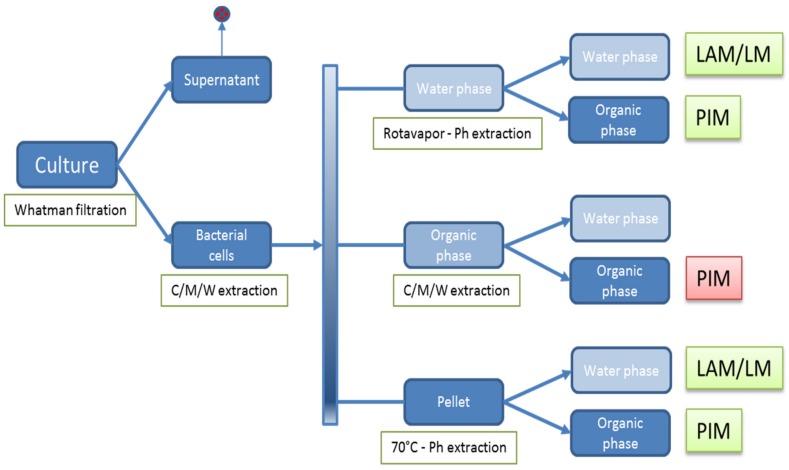
Schematic representation of lipid extraction from mycobacterial cultures. Lipids were extracted from stationary fleece cultures as described in the text. C/M/W: Chloroform/Methanol/Water. Ph: Phenol. LAM/LM: lipoarabinomannan/lipomannan. PIM: phosphatidylinositol mannosides. Green boxes show water-soluble fractions, red boxes indicate water-insoluble fractions.

A schematic representation of the isolation of glycolipids from mycobacterial cultures is presented in Figure 1. Mycobacteria were harvested from the pellicles of stationary cultures or from cultures grown in orbital shaking incubators by a primary separation of the bacterial cells from the cultures using Whatman filtration (Whatman Number 1 filter paper).

Polar lipids were subsequently isolated according to previously published methods [31] as follows. The bacterial cells were subjected to chloroform–methanol–water (CMW) (Merck) (10:10:3 or 10:5:4) extraction, leading to an aqueous phase, an organic phase, and a pellet. The aqueous phase was dried using a Rotavapor^®^ (Buchi, Flawil, Switzerland), the dry material was subjected to a phenol extraction twice using 5 mL of phenol (Merck) and 5 mL of water (Merck) for 30 min at 70 °C. The aqueous phase was washed four times with phenol (Merck) and yielded a water-soluble LAM/LM fraction. The organic phase was washed four times with phosphate buffered saline solution (PBS) and yielded a water-soluble PIM fraction. The organic phase of the bacterial cell extraction was dried under a stream of nitrogen gas. The resulting organic residues were hydrolyzed in 3 mL of 0.01 M HCl for 5 min at 100 °C. Subsequently, PIMs were separated from the saccharide constituents of the polyprenols by the addition of 12 mL of chloroform: methanol (2:1) (Merck) to result in a final CMW ratio of 8:4:3. The organic phase of this extraction yielded a water-insoluble PIM fraction.

Finally, the remaining lower organic phase pellet was dried under a stream of nitrogen gas and extracted twice with 5 mL of phenol (Merck) and 5 mL of water (Merck) for 30 min at 70 °C under constant stirring. The phenol: water extract was cooled and centrifuged at 750 g for 30 min. Following the centrifugation the mixture was biphasic and the aqueous and phenol fractions were collected in separate tubes. The aqueous phase was washed four times with phenol (Merck) and yielded a water-soluble LAM/LM fraction. The organic phase was washed four times with phosphate buffered saline solution (PBS) and yielded water-soluble PIM fractions.

To further separate different PIM species, 5 g of the isolated insoluble fraction was run on a silica gel fractionation column (2.5 cm × 20 cm; Sigma-Aldrich, St. Louis, USA) with a speed of 2 mL/min. The column was eluted stepwise with 5 mL of CMW (ratio ranging from 60:30:0 up to 60:30:8), yielding seven pools of PIM fractions containing PIM fractions of different polarity. These fractions were collected and the content was monitored by thin-layer chromatography (TLC).

### 2.6. Thin-Layer Chromatography Analysis of Lipids

Lipids were analyzed using thin-layer chromatography (TLC, Merck 1.05641). Visualization of resolved lipids was routinely performed using an anisaldehyde staining (versatile stain, staining especially phenols and sugars) consisting of 0.7 mL of p-anisaldehyde in 135 mL of absolute ethanol containing 9.5 mL of concentrated sulfuric acid and 2.7 mL of glacial acetic acid [33]. Occasionally, TLC was stained with cerium ammonium molybdate (CAM), a versatile stain for most functional groups, or ninhydrin stain for amino acids and primary amines. The CAM stain consisted of 2.5 g ammonium molybdate tetrahydrate and 1 g cerium ammonium sulfate dihydrate dissolved in 10 mL sulfuric acid and 90 mL water. The ninhydrin stain contained ninhydrin (0.1 g), acetic acid (0.5 mL) and acetone (100 mL).

### 2.7. Immune TLC

In addition, we developed an immune TLC using bovine serum or monoclonal antibodies. Samples were spotted on a TLC plate and run in a CMW (60:30:6) buffer. Subsequently, the plate was dried and incubated overnight in a petri dish (Greiner Bio-one 688102) in 35 mL n-Hexane (Merck 1.04367) saturated with poly(isobutyl metacrylate) (Sigma-Aldrich 445754), allowing the n-Hexane to slowly evaporate. The following day the TLC was blocked by 1 hr incubation in PBS-1% Bovine Serum Albumin (BSA; Sigma-Aldrich A7906, St. Louis, USA) followed by 1 hr incubation with PBS-1%BSA-0.05% Tween21 (Sigma-Aldrich P2565) and a primary antibody. As primary antibodies we used a 1:100 dilution of bovine serum obtained from an experimentally MB AN5 infected cow or a 1:50 dilution of bovine serum obtained from an experimentally MAP infected cow. Subsequently, the plate was washed three times for 10–15 min each in PBS-1% BSA- 0.05% Tween21 (wash buffer). Plates were then incubated for 1 hr incubation in PBS-1% BSA-0.05% Tween21 (Sigma-Aldrich P2565) and peroxidase conjugated mouse-anti-bovine IgG1 [34] or protein-G-PO (Life technologies), followed by three washes in wash buffer. The TLC was transferred to a clean petri dish and spots were visualized using a 3,3′,5,5′-Tetramethylbenzidine/ dioctylsodium sulphosuccinate(TMB-DONS; (10 mL phosphate-citrate buffer with 100 µL TMB (60 mg/mL, Sigma-Aldrich)—DONS (200 mg/mL, Sigma-Aldrich)) in dimethyl sulfoxide (DMSO; Merck) solution and 6 µL 30% hydrogen peroxide (Merck).

### 2.8. Reference Reagents

Reference reagents used as reference standards on the immune TLC were obtained through BEI Resources; National Institute of Allergy and Infectious Diseases, National Institutes of Health are shown in Table 2.

### 2.9. Recombinant Protein and PPD ELISA

Mature recombinant *M. bovis* protein antigens MPB70 and MPB83 were cloned using *M. bovis* strain AN5 genomic DNA, standard PCR, cloning, and sequencing techniques. The DNA sequence was checked to confirm identity. Mature forms of the MPB70 and MPB83 antigens were expressed as poly-histidine tagged fusion proteins in *Escherichia coli* XL1Blue using the vector pQE80 (Qiagen) and affinity purified. Purity and size were checked using standard SDS-PAGE and Western blot techniques, of which details have been previously described [35].

Ninety-six-well plates (MicroWell^®^ Polysorb^®^, Sigma-Aldrich, St. Louis, USA) were coated with 100 µL/well of one of the following antigens: avian tuberculin PPD (PPD-A, Thermo Fischer, Lelystad, The Netherlands), bovine tuberculin PPD (PPD-B, Thermo Fischer, Lelystad, The Netherlands), *M. bovis* strain AN5 recombinant antigen MPB70, and MPB83. Antigens were diluted to a concentration of 5 µg/mL for PPD-A, PPD-B, MPB70, and to a concentration of 1 µg/mL for MPB83 in 0.05 M carbonate coating buffer (pH 9.6). Antigen coating occurred overnight at 37 °C. After incubation, plates were frozen at −20 °C until further use. On the day tests were performed, plates were thawed at room temperature and wells were emptied and blocked with 200 µL 1% BSA in phosphate buffered saline (PBS, Gibco, USA) at 37 °C with agitation for 1 h to prevent non-specific binding of sample antibodies.

After incubation, the wells were emptied and remaining moisture was removed by tapping the plates on an absorptive paper towel. Serum samples were diluted 1:20 into a test buffer containing 1% BSA in PBS with 0.05% Tween 20 (Merck, Darmstadt, Germany) and mixed by pipetting. A 100 µL sample dilution was added per well and plates were incubated at 37 °C with agitation for 1 h. Next, plates were washed two times with demineralized water and 0.05% Tween 20 and moisture was removed. Then, 100 µL recombinant protein G-HRP conjugate (Invitrogen, Waltham, USA) diluted 1:32,000 in 1% BSA with 0.05% Tween 20 and 0.5 M NaCl (Sigma-Aldrich, St. Louis, USA) buffer was added and plates were incubated at 37 °C with agitation for 1 h. After incubation, washing and drying procedures were repeated and 100 µL tetramethylbenzidine (TMB) substrate (Diarect AG, Freiburg im Breisgau, Germany) was added per well and incubated for 15 min at room temperature. The reaction was stopped by adding 100 µL 1 M H_2_SO_4_.

Responses, expressed as optical densities (OD), were measured at 450 nm with a 635 nm reference filter (EL 808 Ultra micro plate reader, Bio-Tek instruments, Winooski, USA). On each plate, positive and negative control sera were included and used to correct for inter-plate variation. Results were expressed as sample to positive ratios (S/P), calculated as the OD value of the sample − the OD value of the positive control.

### 2.10. PIM ELISA

Fractions containing water-soluble PIM failed to coat to various types of 96-well plates suitable for ELISA (data not shown). The C/M/W fraction containing water insoluble PIM (Figure 1, red box) was dried weighed and reconstituted in methanol at 10 mg/mL as a stock solution. A 5 µg/mL PIM/methanol solution was prepared from the stock solution and was added to microplate wells (100 µL per well in 96 well plates (Nunc-Immuno™ MicroWell™ 96 well PolySorp^®^ flat bottom plate, Sigma-Aldrich, Zwijndrecht, The Netherlands). The methanol was allowed to evaporate overnight. Wells were subsequently blocked using 200 µL per well of PBS containing 1% BSA (Sigma-Aldrich, St. Louis, USA) for 60 min at 37 °C on a 600 rpm orbital shaking platform. Blocking buffer was removed and bovine serum (1:20 dilution in blocking buffer) was incubated for 60 min at 37 °C on a 600 rpm orbital shaking platform. Plates were washed four times in 0.5% PBS with 0.05% T20, and subsequently the peroxide labelled mouse-anti-bovine IgG (Life technologies), mouse-anti-bovine IgG1, mouse-anti-bovine IgG2 [34], or protein-G-PO (Life technologies) was added as indicated and incubated for 60 min at 37 °C on a 600 rpm orbital shaking platform. Plates were incubated at room temperature for 15—30 min. The reaction was stopped by adding 100 µL of 1.5 M sulfuric acid to each well. The absorbance of each well was measured at 450 nm and background corrected OD450 values are shown.

When stated, the PIM specific ELISA was expanded to an absorbed ELISA using the provided pre-absorption buffer described above. Colostrum and sera were incubated with the commercially available pre-absorption buffer (ID-Vet) according to the provided manufacturer’s instructions. Subsequently, the colostrum and sera were tested in the PIM ELISA at dilutions similar to the original PIM ELISA.

### 2.11. Antigenic Structures in PIM

Polysorb ELISA plates were coated with insoluble PIM enriched fractions. To degrade protein components in these fractions, they were treated with 0.3% protein-K (Prot-K) dissolved in PBS, as described previously [36]. To oxidize carbohydrate moieties, PIM enriched fractions were treated with 50 mM sodium periodate (PI) in 0.1 M NaAc (pH 5), as described [18]. Subsequently, ELISAs were performed as described above.

### 2.12. Statistics

The PIM preparations from MAP 316F and MB AN5, which were both treated with Prot-K and Na-PI, were analyzed using ELISA with 16 different sera. The blank corrected OD405 data were analyzed using a paired Student t-test between the native PIM and the Prot-K treated PIM, as well as the native PIM and the Na-PI treated PIM. Level of significance was set at *p* < 0.05 (Figure 4).

ELISA data comparing responses to tuberculins (PPDB, PPDA), recombinant antigens (MPB70, MBP83), and PIM-HCl were analyzed using ANOVA at antigen level with Bonferroni correction for multiple comparisons, as indicated. (Figure 6). Level of significance was set at *p* < 0.05.

Immunoglobulin isotype IgG1 and IgG2 specific ELISA data were not formally statistically analyzed, as the data indicated that on infection status level the longitudinal lines crossed at one or more time points, indicating no difference at the level of infection status (Figure 7). The ELISA data regarding the maternal transfer of PIM specific antibodies were not formally statistically analyzed, as the data were used to illustrate the maternal transfer of antibodies from dam to calf and formal statistical analysis did not add additional clarity to these observations.

Data visualization and statistical analysis was performed using GraphPad Prism version 8.3.

## 3. Results

### 3.1. Extraction of Polar GLs and PIM Detection with an Improved Immune TLC

To optimize the visualization of PIM species, an immune TLC was developed using experimentally MAP or MB infected serum as the primary antibody. Runs with purified reference lipid preparations isolated from *M. tuberculosis* showed that specific reactivity to PIM 1, 2, and 6 molecules was achieved using this method (Figure S1). Besides this, more discrete spots were detected compared to the anisaldehyde staining, indicating that the immune TLC achieved higher resolution and was more sensitive in identifying PIM species. The improved sensitivity revealed that in certain supposedly highly purified lipid preparations, traces of highly immune reactive PIM 1 and 2 species were consistently present, which were not detected with conventional anisaldehyde staining (Figure S1).

**Figure 2:**
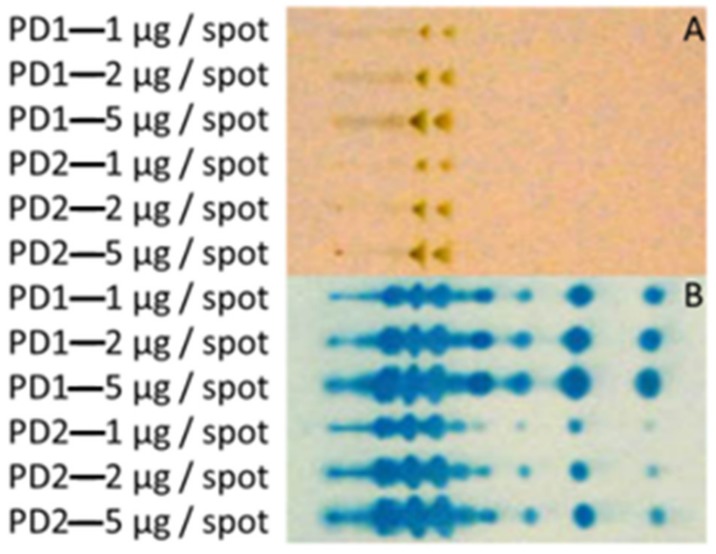
(Immune) TLC of different phosphatidylinositol mannosides (PIM) enriched water-soluble fractions derived from *Mycobacterium bovis* (MB) AN5. PD1 samples were isolated from shaken cultures, PD2 were extracted from stationary pellicles. Totals of 1, 2 and 5 µg were spotted of each sample. Anisaldehyde staining (**A**) and immune TLC using MB infected cattle serum as primary antibody (**B**) were used for visualization. Samples were spotted on the left side of the TLC.

The developed immune TLC was used to visualize the extracted GL from cellular fractions. This confirmed that PIM extraction was successful, as various PIM species were present in the fractions (Figure 2). Moreover, immune staining indicated that a high number of components in the PIM enriched extract are dominant targets for antibodies present in the serum of a cow with experimental MB infection. Attempts to separate the individual PIMs from the extracts were unsuccessful, although a shift from apolar to more polar PIM species was observed in fractions with increasing water concentrations (Figure S2). Apparently, the physical–chemical properties of the individual PIMs were too similar to allow further purification.

### 3.2. Cross-reactions of Immune Sera from MAP and MB Infected Cows

**Figure 3:**
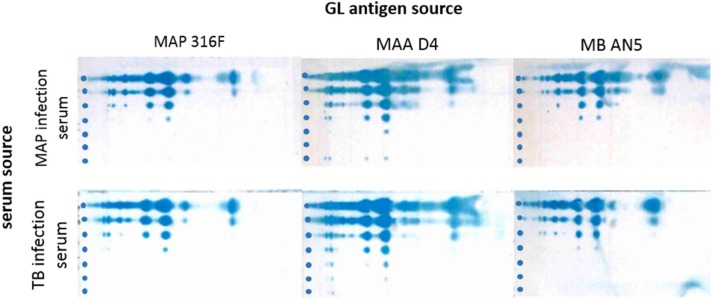
Visualization of cross reactions on both antigen and antibody level between *Mycobacterium avium* subsp. *paratuberculusis* (MAP), *Mycobacterium avium* subsp. *avium* (MAA), and *Mycobacterium bovis* (MB) using immune TLC. The immune TLC was performed using experimental MAP or MB infected sera and visualized using protein-G-PO and TMB-DONS staining. As antigen source, 2 µL of the first organic phase extract from MAP 316F, MAA D4, or MB AN5 was used, starting at a concentration of 100 µg/mL (upper lane) with a stepwise tenfold serial dilution for each subsequent spot. Samples were spotted on the left side of the TLC and are indicated by dots.

Lipids isolated from the MAP strains 316F, MAA D4, and MB AN5 were stained in immune TLC using sera from cows infected with MAP and MB. The results indicated that the sera cross-reacted in both ways, as is exemplified in Figure 3. Hence, PIMs isolated from three mycobacterial species have immunodominant B cell epitopes to which generic, species-unspecific antibodies are generated in both MAP and MB infected hosts.

### 3.3. Development and Characterization of the PIM ELISA

To further explore the apparent immunodominant nature of the PIMs with respect to antibody responses in cattle with MB or MAP infection, an ELISA was developed using the PIM enriched water-soluble and water-insoluble fractions as coating. Based on the comparison of reactions on different types of microtiter plates and conditions, the PIM enriched water-insoluble fraction performed best on Nunc polysorb microtiter plates. Plates were coated with 5 µg/mL of the C/M/W PIM extract (Figure 1 red box) in methanol, and dried overnight at room temperature.

**Figure 4:**
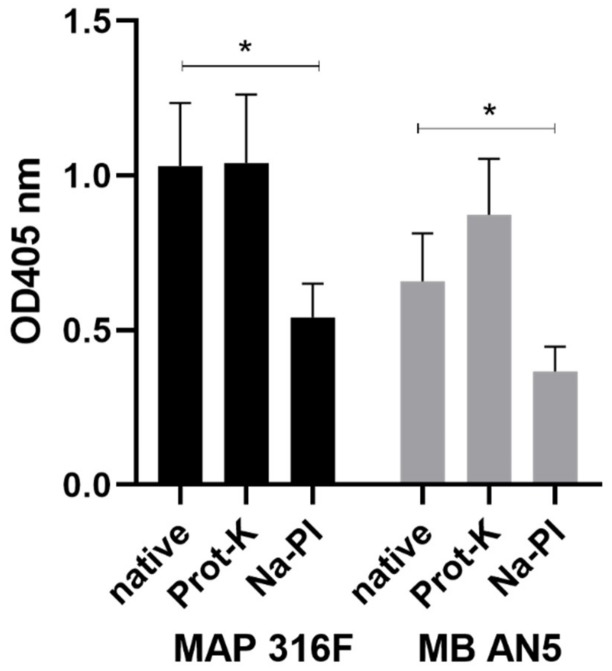
The immunodominant B cell epitopes on PIM are insensitive to Prot-K treatment and sensitive to Na-periodate treatment. PIM ELISAs were coated with the PIM enriched water-insoluble fractions from MAP 316F (left) and MB AN5 (right). Subsequently, the PIM fractions were subjected to Prot-K treatment or Na-periodate treatment and compared to untreated fractions. The binding of IgG1 antibodies to PIM was measured using serum from experimentally MAP infected animals and expressed as blank corrected optical density values. Results are expressed as the mean + SEM from triplicate experiments. Asterisks denote significant differences (*p* < 0.05) between means of the native and Na–PI treated group as determined with Student’s t test.

To exclude the possibility that potential protein contaminants in the PIM fractions reacted with serum antibodies in the ELISA, the PIM enriched water-insoluble fraction was subjected to Prot-K treatment after coating to remove protein components. Prot-K treatment of PIM preparations did not alter antibody binding significantly, indicating that protein components play a minor role with respect to immunodominant B cell epitopes in PIM fractions (Figure 4). Treatment of PIM fractions with Na-periodate treatment (oxidates carbohydrate moieties) resulted in a loss of signal. This demonstrates that carbohydrate moieties are important in the binding of the IgG1 antibodies to PIM (Figure 4). Results were comparable for PIM enriched fractions originating from the MB AN5 strain or the MAP 316F strain when probed with serum from experimentally MAP infected animals.

### 3.4. Effects of Skin Test and Mycobacterial Exposure on PIM Specific Antibody Responses in Cattle

**Figure 5:**
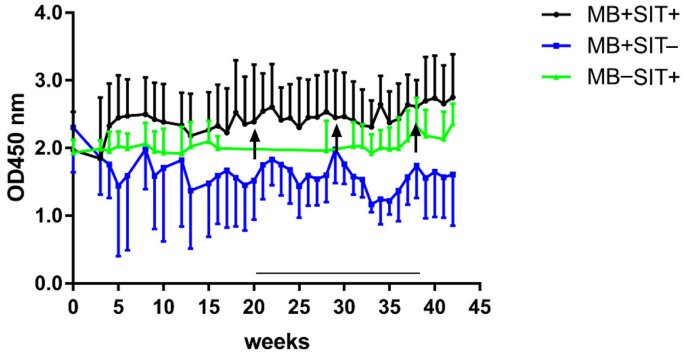
Experimental MB infection and SIT did not significantly alter antibody responses against PIM in cattle. Serum of MB infected cattle was examined with PIM ELISA. The study consisted of three groups of calves, aged 6 months at the start of the experiment. Calves in group MB + SIT + (*n* = 5) and MB + SIT − (*n* = 4) were experimentally infected with MB through IV injection of 1 mg of MB strain AN5. Group MB − SIT − (*n* = 5) served as uninfected controls. Groups MB + SIT+ and MB − SIT + were skin tested with a single intradermal test (SIT) (3000 IU PPD-B) at days 20, 32, and 40 post infection (MB + SIT +) or at the start of the experiment (MB − SIT +). Arrows point at days of SIT tests in group MB + SIT +. Data are expressed as the group mean background corrected optical density. Error bars represent the SEM.

The PIM ELISA was used to screen stored sera from experimentally MB infected cattle (Table 1, group 4) for antibody presence. The study consisted of three groups of calves, aged approximately 6 months at the start of the experiment. Calves in group MB + SIT + (*n* = 5) and group MB + SIT − (n = 4) were experimentally infected with MB through IV injection of 1 mg of MB strain AN5. Group MB − SIT + (*n* = 5) served as uninfected control (Table 1 group 5). Groups MB + SIT + and MB − SIT + were skin tested using the single intradermal test (SIT) with PPD-B (3000 IU) at weeks 20, 32, and 40 post infection (MB + SIT +) or at the start of the experiment (MB − SIT +). As can be observed in Figure 5, antibody responses to the PIM enriched fraction were high in all groups at the start of the experiment, remained high irrespective of treatment, and were not consistently influenced by SIT skin testing.

**Figure 6:**
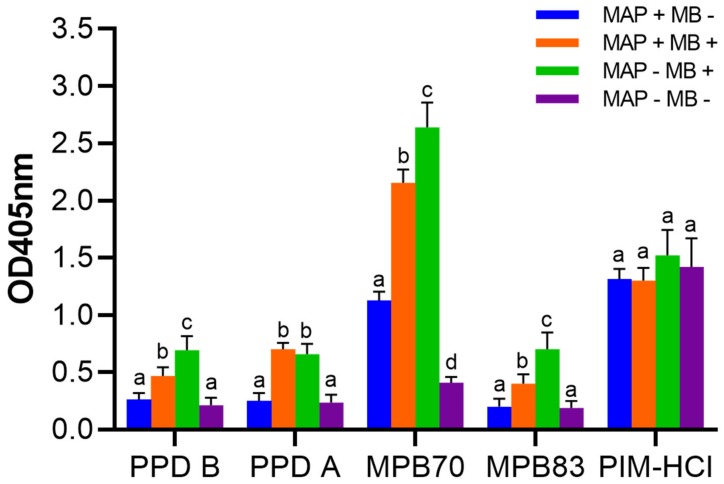
Serological responses to PIM antigens in comparison to reactions against PPD-A and PPD-B tuberculin, and the MB MPB70 and MPB83 *E. coli* recombinant proteins. Sera were tested from four different groups of adult cattle defined by infection status (group 6–9 in Table 1). Blue: MAP positive and MB negative animals (MAP + MB −), *n* = 254. Orange: MAP positive and MB positive animals (MAP + MB +), *n* = 147. Green: MAP negative and MB positive (MAP − MB +), *n* = 69 animals. Purple: MAP negative and MB negative animals (MAP − MB −), *n* = 30 animals. Data are expressed as the group mean background corrected optical density. Error bars represent the SEM. Data were analyzed using ANOVA at antigen level. Different letters indicate a significant difference between conditions (*p* < 0.05).

Next, we tested a panel of cross-sectional serum samples from adult cows with a different status of mycobacterial infection (MAP or MB) for reactivity to PIM antigens and compared these to reactions against ELISAs based on PPD-A and PPD-B, and the MB MPB70 and MPB83 *E. coli* recombinant proteins. We tested sera from four different groups of animals defined on infection status (Table 1, group 6, 7, 8, 9): MAP positive animals and MB negative (MAP + MB −), *n* = 254; MAP positive and MB positive animals (MAP + MB +), *n* = 147; MAP negative and MB positive (MAP − MB +), *n* = 69 animals; and MAP negative animals and MB negative animals (MAP - MB -), *n* = 30 animals. While at group level differences between the reaction against PPDs and the recombinant MB antigens MPB70 and MPB83 could be observed, the sera obtained from the cows in the various infection status groups showed similar antibody reactions to PIM (Figure 6). Hence, the MAP or MB infection status as well as skin testing did not affect the PIM antibody response.

### 3.5. Effect of Age and M. Avium Subsp. Paratuberculosis Infection Status on PIM Reactivity

**Figure 7:**
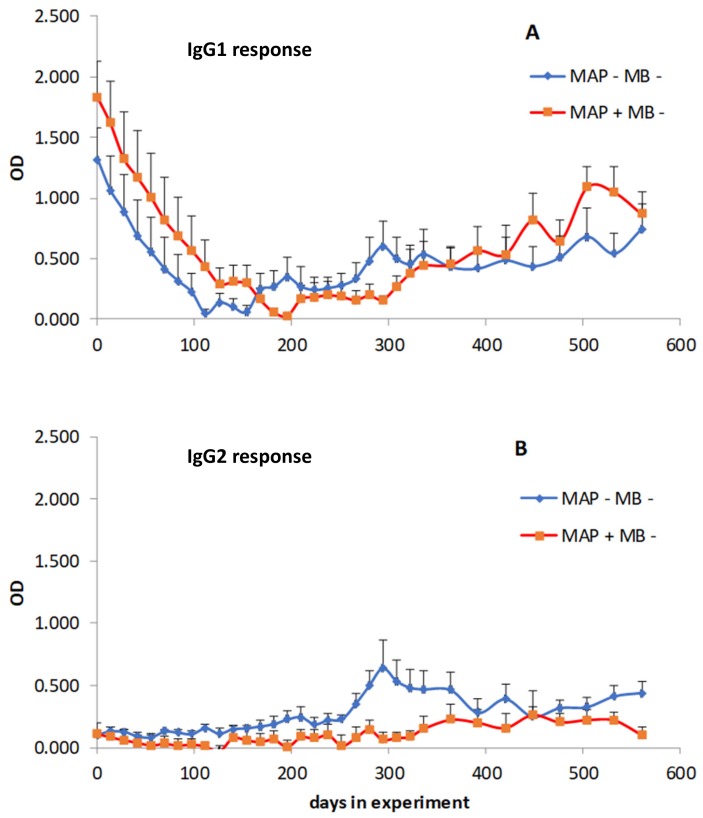
IgG1 and IgG2 isotype distribution of PIM specific antibodies was similar between MAP infected and control animals, with a strong bias towards IgG1 responses. Sera obtained from calves sourced from a certified MB free farm experimentally infected with MAP during the first month of life (MAP + MB −) were used for a PIM ELISA. As controls, blood samples from 10 calves from certified MB and MAP unsuspected (MAP − MB −) herds were taken monthly starting in the first week of life for a period of 18 months as part of a larger longitudinal follow-up study. The presence of PIM specific IgG1 (**A**) and IgG2 (**B**) antibodies was tested. Results are expressed as the group mean background corrected optical density. Error bars represent the SEM.

The cows used in Section 3.4 were conventional cows obtained from MB free Dutch herds with unknown herd infection status for paratuberculosis, which entered the study at 6 months of age. To exclude that the initial high PIM responses originated from exposure to MAP, a second set of sera was screened in the PIM ELISA. Blood samples from 10 control calves sourced from herds with a certified paratuberculosis unsuspected (status 10) and MB free status were tested for seroreactivity against PIM. These samples were compared to blood samples taken from 10 calves which were sourced from MB free herds and experimentally infected with MAP during the first month of life (MAP infected). The two groups showed similar IgG1 and IgG2 isotype specific PIM antibody responses with a clear bias to IgG1 responses, as illustrated in Figure 7, which was independent of MAP infection status. These results suggest that maternal transfer of antibodies may play an important role, especially regarding the IgG1 isotype responses, as high initial levels of PIM specific IgG1 antibodies were detected that rapidly declined during the first months of life.

To further examine the suspected maternal transfer of PIM specific IgG1 antibodies via colostrum, calves born to cows in the above study were examined. A set of serum samples was analyzed, which consisted of serum samples of the dam on the day of calving, a serum sample from the calf directly after birth prior to the first colostrum feeding, a colostrum sample as it was fed to the new-born calf, and a subsequent serum sample from the calf two to seven days following colostrum uptake. Subsequently, during the first six months four additional samples were analyzed. Results revealed that the PIM specific antibodies are present in colostrum, while serum levels in the dams are relatively low at that time (Figure S3A). Serum samples taken from calves directly after birth, prior to colostrum uptake, did not contain measurable amounts of PIM specific antibody. The PIM specific antibodies were transferred to the majority of calves following colostrum feeding. During the first six months of life the level of PIM specific antibodies in sera taken from these calves waned, which corresponds to the passive, maternal transfer of antibodies via colostrum.

**Figure 8:**
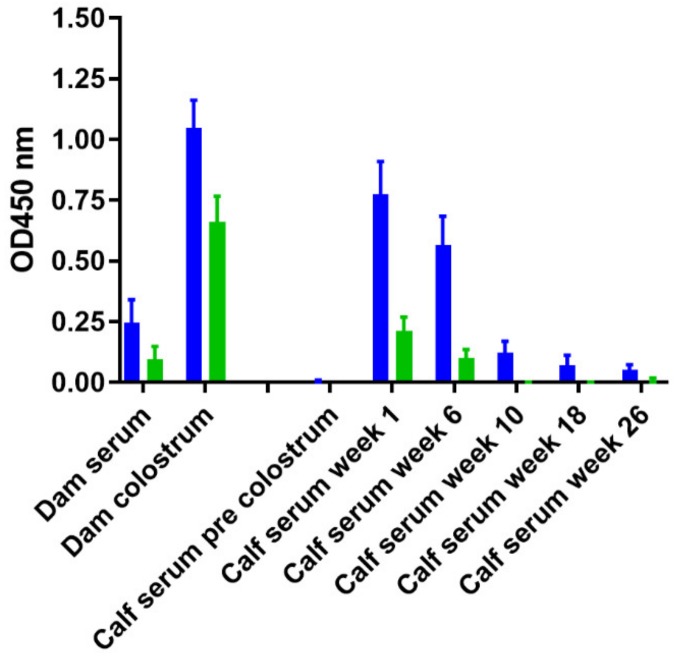
Maternal transfer of PIM specific antibodies from dams to calves through colostrum. PIM specific antibody levels were measured in the colostrum and serum of dams, and in the serum of calves born from these dams and expressed as blank corrected optical density. Serum and colostrum from the dam were taken on the day of parturition. Serum from the calves was obtained at indicated time points and treated with a commercially available absorption buffer (ID-VET) used in an absorbed MAP ELISA according to instructions provided. Data are represented in a comparative format and shown as group average + SEM. Blue bars represent pre absorption values, while green bars show the PIM antibody level after absorption.

In the serodiagnostics of MAP infection, an absorbed ELISA format is commonly used. In this type of ELISA the serum (or milk) is initially diluted in a pre-absorption buffer. The pre-absorption buffer contains soluble antigens from non-pathogenic mycobacteria, typically *M. phlei*. We expanded our PIM specific ELISA to an absorbed ELISA using an absorption buffer from a commercially available diagnostic absorbed MAP ELISA kit (ID-Vet). Colostrum and sera were incubated with the absorption buffer and subsequently tested using the PIM ELISA at dilutions similar to the original PIM ELISA (Figure S3A,B). PIM antibody levels reduced significantly after incubation with absorption buffer (Figure 8 and Figure S3C), and we observed that approximately 80% of the PIM specific reactivity could be absorbed from serum samples in both dams and calves (Figure S3C). The efficiency of absorbing PIM specific antibodies in colostrum was markedly less (approximately 55%).

### 3.6. The Presence of PIM in Commonly used Mycobacterial Antigen Preparations

**Figure 9:**
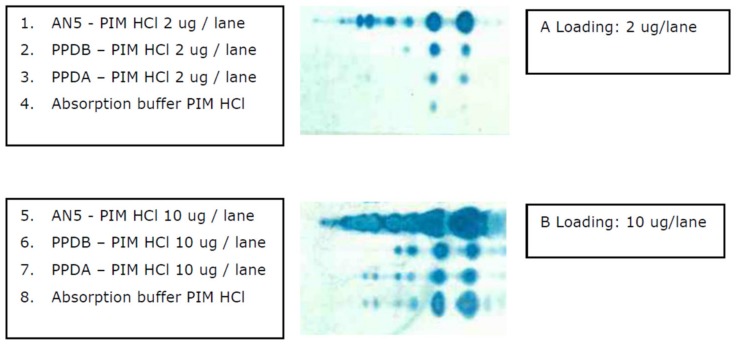
PIMs are present in commonly used mycobacterial antigen preparations. Polar glycolipids were extracted from commercially available tuberculins, PPD-A and PPD-B. In addition, polar glycolipids were extracted from an absorption buffer from a commercially available MAP absorbed ELISA (ID-Vet). Polar glycolipids extracted from MB AN5 were used as reference. Panel A shows the result of the immune TLC ran with 2 µg antigen per spot and visualized using serum derived from a MB infected cow as primary antibody. Panel B is similar to Panel A, however 10 µg per antigen spot was used for the TLC. Samples were spotted on the left side of the TLC.

We demonstrated that IgG1 antibodies to PIM antigens are common in young calves and dairy cows irrespective of mycobacterial infection (MAP/MB) status, and that PIM antibodies have targets in absorption buffers. Subsequently, we examined sources of mycobacterial antigens commonly used in antibody based diagnostic assays. We used PPD-A and PPD-B tuberculins as standardized antigens used in diagnostic tests. Results show that PIMs are present in commonly used mycobacterial antigen preparations, such as PPDs and buffers used to absorb non-specific signals from sera in absorbed-ELISA formats used for serodiagnostics of ruminant paratuberculosis. Based on the immune TLC, as shown in Figure 9, the major representatives were the PIM1 and two glycolipid antigens. These results suggest that PIM antigens are non-specific antigens with ubiquitous immunodominant B cell epitopes that cross-react with PIMs from other mycobacterial species. Therefore, PIMs could reduce the specificity of diagnostic tests when crude extracts are used as antigen source, and PIMs are not properly filtered out.

## 4. Discussion

In the current study, we aimed to determine the potential role of PIMs as antigens with diagnostic value in serological assays in order to improve the accurate diagnosis of mycobacterial infections in cattle. To identify the different PIM species, a novel immunological TLC was developed based on GL binding to antibodies present in bovine serum derived from MB or MAP infected cows. A high number of components in the PIM enriched extract were dominant targets for antibodies present in infected cow serum, and PIMs were more clearly visualized with this method compared to other conventional TLC techniques. Therefore, the developed immune TLC provided a more sensitive approach in PIM detection.

PIM responses detected by the immune TLC and by PIM coated ELISAs were prevalent in the tested cows. The majority of bovine hosts generated specific IgG class antibodies against PIM, but this was unrelated to mycobacterial infection status or age. Skin testing MB infected animals, which has been shown to increase antibody responses to complex and protein antigens [37,38], did not lead to enhanced PIM specific responses either. The antibodies present in MB infected serum also reacted with *M. tuberculosis* reference lipids, indicating the existence of highly conserved epitopes in PIMs. Therefore, our results suggest that immunological PIM responses are non-specific, and that cross-reactions occur due to common antigenic determinants on PIM from MAP and MB, but also from other mycobacteria. Similar cross-reactions have been observed in sera from patients with leprosy, where IgG antibodies reacted to *M. tuberculosis* PIMs due to shared L4-PIM epitopes [39]. IgG antibodies to L4-PIM were also detected in healthy controls, regardless of their immune status [40]. This indicates that PIMs are common immunodominant mycobacterial antigens that evoke a non-specific IgG immune response.

The exact PIM epitopes recognized by antibodies are unknown, but our data show that the carbohydrate moiety of PIM contributes substantially to antibody binding. A monoclonal antibody isolated in a previous study also required the PIM mannoside residue for efficient binding [39]. For other antigenic GLs, such as LAM, an important immunodiagnostic MB and MAP target [29,41], heterogeneous epitopes have been identified dependent on the type of capping. In pathogenic species, the arabinomannan is capped by mannose [15], and both arabinan and mannose appear to be antigenic determinants recognized by antibodies [42]. This suggests that the immunological determinants on polar GLs are, at least partly, associated with the mannose moieties of these GLs.

Part of the observed PIM immune responses, especially in calves up to six months of age, were derived from passive transfer following colostrum uptake. The specificity of commercial serological MAP tests is influenced by days in milk, and studies have shown that colostrum contains high concentrations of non-specific antibodies. Therefore, the chance of non-specific ELISA reactions is higher in colostrum compared to milk sampled at later stages, as high concentrations of non-specific proteins in colostrum enhance antibody binding [43,44]. However, in our experiment a maternal derived antibody pattern was observed in serum as well, confirming the passive transfer of PIM antibodies by colostrum. Since there is no clear evidence in the data that (experimental or natural) exposure to either MB or MAP induces or stimulates the levels of PIM specific antibodies, a major remaining question relates to the nature of the exposure and immune stimulation that leads to the priming of animals and the strong accumulation of these antibodies in colostrum, especially since the serum of dams contained relatively low PIM antibody concentrations.

PIMs were also identified in the antigenic preparations PPD-A and PPD-B. These extracts are known to encompass cross-reactive components that inhibit test specificity [7]. Several cross-reactive proteins were identified [45,46], but the nature of most cross-reactive components is unknown. Since antibodies are non-specifically generated against PIMs, this indicates they are cross-reactive agents that impair serological test specificity. To enhance the specificity of serological tests, a pre-absorption step was used to filter out the majority of cross-reactive agents by incubation with an absorption buffer. Indeed, immune TLC showed that the *M. phlei* absorption buffer comprised PIM antigens to which PIM antibodies present in MB infected serum bind. Although pre-absorption greatly improves the specificity of ELISAs, false-positive results have still been reported in areas with high prevalence of environmental bacteria, including southern and south-eastern United States [47]. This indicates that the occurrence of false-positives and concordant test specificity greatly depends on location.

For T-cell based diagnostics, the single intradermal comparative cervical tuberculin (SICCT) test is often employed to detect bovine tuberculosis. In this test, skin reactions to PPD-A to PPD-B are compared to increase specificity. However, the comparison to PPD-A may not be appropriate in animals or regions where colonizing or sensitizing NTMs are only distantly or not at all related to *M. avium* [7]. Moreover, the sensitivity and specificity of the SIT and SICCT could be affected in MAP infected herds [48]. Therefore, our results suggest that PIMs generate a non-specific immune response that inhibits mycobacterial diagnostic test specificity when using complex antigen preparations.

Although the humoral PIM response is too unspecific for the implementation in diagnostic assays, the CD1 based T-cell response to lipid extracts of naturally infected MAP and MB infected animals is highly specific, and exclusively detected in infected animals [36]. Among these lipids, PIMs, and specifically AcPIM_6_, are recognized by CD3 receptors, resulting in natural killer T-cell activation and induced interferon gamma production in bovine lymphocytes [48]. This suggests that PIMs elicit a specific cell mediated immune response, and that PIMs could be suitable antigen candidates for novel (non-classical) T cell-based tests.

## 5. Conclusions

Antibody-based tests using PIMs provided evidence that specific IgG class antibodies are generated against these antigens in the majority of bovine hosts. However, this is irrespective of mycobacterial infection status, which makes these antigens unsuitable targets for novel antibody-based indirect diagnostic assays. We also conclude that mycobacterial polar PIMs that are present in commonly used antigenic preparations (such as PPD) can limit the diagnostic specificity of mycobacterial serological assays. Serum pre-absorption buffers have been shown to greatly increase test specificity in diagnostic MAP ELISAs based on crude MAP antigens, and we reveal that these buffers effectively bind PIM specific antibodies, thereby limiting cross-reactive responses in these assays.

## Figures and Tables

**Table 1:** Overview of serum samples analyzed.

Number	Infection Status (*)	Experimental or Natural Infection	Number of Cows	Serum Samples (n)	Duration of Study	Reference
1	MAP + MB −	experimental	20	700	4 years	[30]
2	MAP + MB −	experimental	10 + 10 ^#^	350	2 years	[31]
3	MAP – MB −	n/a (controls)	10 + 10 ^#^	350	2 years	[31]
4	MAP − MB +	experimental	9	120	20 weeks	unpublished
5	MAP − MB −	n/a (controls)	5	120	20 weeks	unpublished
6	MAP − MB +	natural	69	69	Diagnostic	Serum biobank
7	MAP − MB −	n/a (controls)	30	30	Diagnostic ^%^	Serum biobank
8	MAP + MB −	natural	254	254	Diagnostic	Serum biobank
9	MAP + MB +	natural	147	147	Diagnostic	Serum biobank

*: The infection status of the animals in group was determined by (repeated) bacterial culture, PCR and (histo) pathology; + indicates infection, − indicates test negative. MAP: *Mycobacterium avium* subsp. *paratuberculosis*, MB: *Mycobacterium bovis*. ^%^: The infection status of group 7 (MAP − MB −) was determined using farm history (no history of cases, no trading of animals), farm topographical isolation, and repeated testing for both MAP and MB. ^#^: Calves born to these cows were sampled prior to first colostrum feeding and up to 26 weeks of life.

**Table 2:** Reference standards used on the immune TLC.

Number	Reagent	Species of Origin
NR-14846	Purified Phosphatidylinositol mannosides (PIM) 1 and 2 H37Rv	*M. tuberculosis*
NR-14844	Purified Trehalose Dimycolate H37Rv	*M. tuberculosis*
NR-14845	Purified Sulfolipid-1 H37Rv	*M. tuberculosis*
NR-14847	Purified Phosphatidylinositol mannosides (PIM) 6 H37Rv	*M. tuberculosis*
NR-14848	Purified Lipoarabinomannan (LAM) H37Rv	*M. tuberculosis*
NR-14850	Purified Lipomannan (LM) H37Rv	*M. tuberculosis*
NR-20328	Purified dimycocerosate H37Rv	*M. tuberculosis*
NR-48784	Purified trehalose monomycolate (TMM) H37Rv	*M. tuberculosis*

## Supplementary Materials

The following are available online at https://www.mdpi.com/2306-7381/6/4/91/s1, Figure S1. (Immune) TLC of reference lipid fractions. TLC on eight lipid reference fractions isolated from *M. tuberculosis* (2 µg/spot). After running, spots were visualized with serum derived from an experimentally MB infected cow as primary antibody in immune TLC and Protein-G-PO (**A**) or with anisaldehyde staining (**B**). Samples were spotted on the left side of the TLC. Figure S2. (immune) TLC on lipid fractions enriched for water insoluble PIM and fractions from a silica column eluted with C/M/W with increasing amounts of water (A1–A7). The lipids were isolated from *M. bovis* AN5 and run with 2 µg/spot on immune TLC and visualized with Protein-G-PO (**A**) or TLC with anisaldehyde staining (**B**). Samples were spotted on the left side of the TLC. Figure S3 **A.** PIM specific antibody levels were measured in colostrum and serum of dams, and in serum of calves born from these dams, and expressed as blank corrected optical density. Serum and colostrum from the dam were taken on the day of parturition. Serum from the calves was obtained at indicated time points. **B.** Colostrum and sera were incubated with a commercially available absorption buffer (ID-Vet) used in an absorbed MAP ELISA according to instructions provided. Subsequently, the colostrum and sera were tested in the PIM ELISA at dilutions similar to the original PIM ELISA. **C.** Reduction in PIM specific signal when comparing the original signal in the PIM ELISA (A) with the signal in the absorbed ELISA (B) and expressed as the percentage reduction of signal. Panel C**#**: calf serum pre colostrum showed no PIM signal, so signal reduction could not be calculated.
