# Supplementary material for: The Role of Phosphatidylinositol Mannosides in the Serological Diagnosis of Mycobacterial Infections
Source: Vet Sci. 2019 Nov 13;6(4):91. doi: 10.3390/vetsci6040091 (PMC6958488; doi:10.3390/vetsci6040091)
Supplement: Supplementary file 1 [file vetsci-06-00091-s001.pdf]

veterinary sciences

MDPI

Article

The Role of Phosphatidylinositol Mannosides in the Serological Diagnosis of Mycobacterial Infections

Ad P. Koets [1,2,*], Marielle H. van den Esker [1], Karel Riepema [1] and Douwe Bakker [3]

[1] Department of Bacteriology and Epidemiology, Wageningen Bioveterinary Research, Houtribweg 398221 RA Lelystad, The Netherlands; marielle.vandenesker@wur.nl (M. H. v.d. E.); karel.riepema@wur.nl (K. R.)
[2] Department of Farm Animal Health, Faculty of Veterinary Medicine, Utrecht University, Yalelaan 73584 CL Utrecht, The Netherlands
[3] Independent Researcher, 8212 AM Lelystad, The Netherlands; douwe.bakker@kpnmail.nl
* Correspondence: ad.koets@wur.nl

Received: 30 September 2019; Accepted: 8 November 2019; Published: date

Supplementary Materials

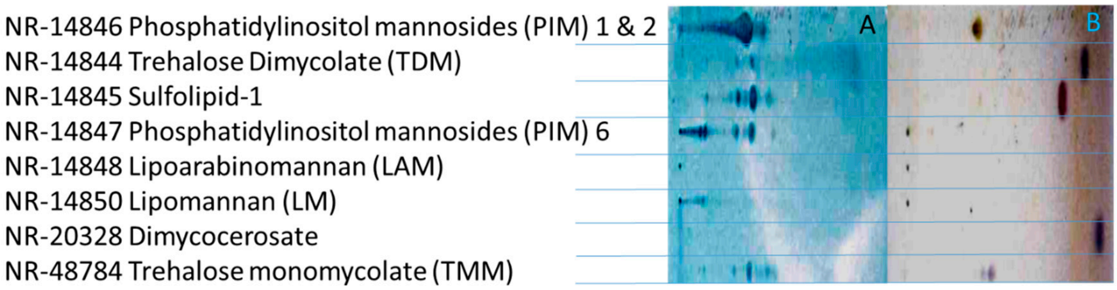

Figure S1. (Immune) TLC of reference lipid fractions. TLC on eight lipid reference fractions isolated from *M. tuberculosis* (2 µg/spot). After running, spots were visualized with serum derived from an experimentally MB infected cow as primary antibody in immune TLC and Protein-G-PO (**A**) or with anisaldehyde staining (**B**). Samples were spotted on the left side of the TLC.

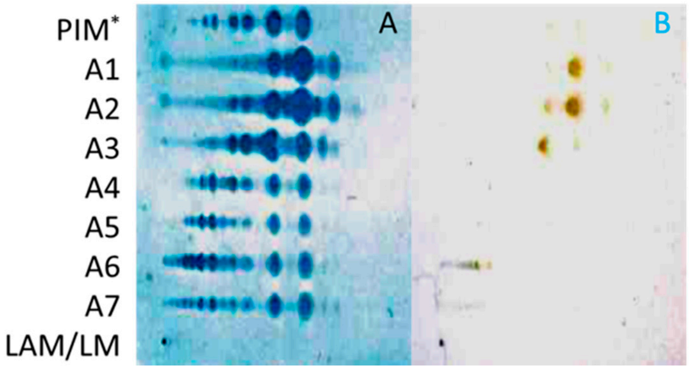

Figure S2. (immune) TLC on lipid fractions enriched for water insoluble PIM and fractions from a silica column eluted with C/M/W with increasing amounts of water (A1–A7). The lipids were isolated from *M. bovis* AN5 and run with 2 µg/spot on immune TLC and visualized with Protein-G-PO (**A**) or TLC with anisaldehyde staining (**B**). Samples were spotted on the left side of the TLC.

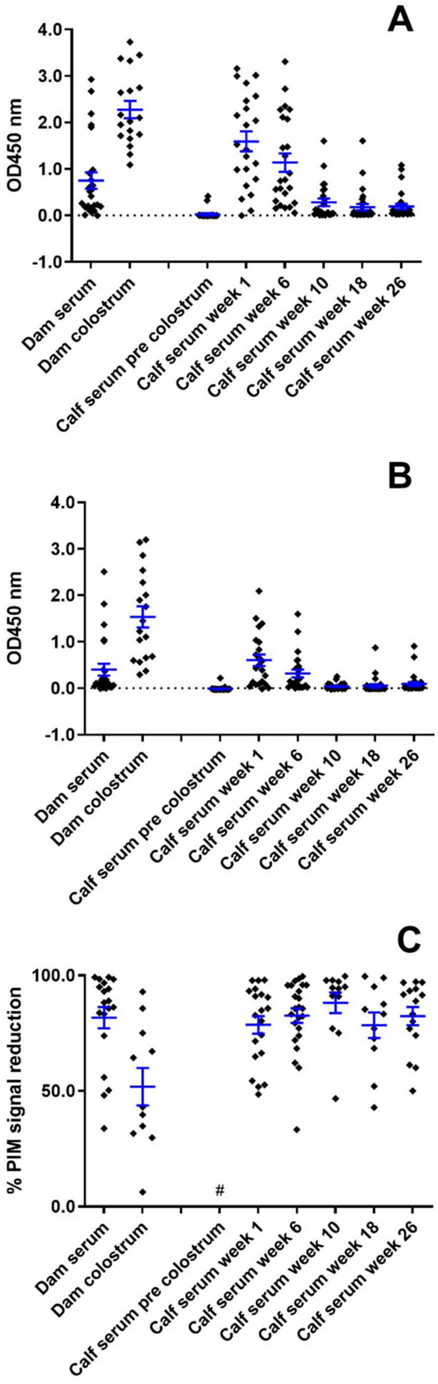

Figure S3. PIM specific antibody levels were measured in the colostrum and serum of dams, and in the serum of calves born from these dams, and expressed as blank corrected optical density. Serum and colostrum from the dam were taken on the day of parturition. Serum from the calves was obtained at indicated time points. **B.** Colostrum and sera were incubated with a commercially available absorption buffer (ID-Vet) used in an absorbed MAP ELISA according to instructions provided. Subsequently, the colostrum and sera were tested in the PIM ELISA at dilutions similar to the original PIM ELISA. **C.** Reduction in PIM specific signal when comparing the original signal in the PIM ELISA (A) with the signal in the absorbed ELISA (B) and expressed as the percentage reduction of signal. Panel **C#**: calf serum pre colostrum showed no PIM signal, so signal reduction could not be calculated.
